# Neoadjuvant anthracycline-based (5-FEC) or anthracycline-free (docetaxel/carboplatin) chemotherapy plus trastuzumab and pertuzmab in HER2 + BC patients according to their TOP2A: a multicentre, open-label, non-randomized phase II trial

**DOI:** 10.1007/s10549-024-07285-y

**Published:** 2024-03-07

**Authors:** Angeline Ginzac, Ioana Molnar, Xavier Durando, Thibault De La Motte Rouge, Thierry Petit, Véronique D’hondt, Mario Campone, Nathalie Bonichon-Lamichhane, Laurence Venat Bouvet, Christelle Levy, Paule Augereau, Barbara Pistilli, Olivier Arsene, Christelle Jouannaud, Suzanne Nguyen, Anne Cayre, Lucie Tixier, Céline Mahier Ait Oukhatar, Jean-Marc Nabholtz, Frédérique Penault-Llorca, Marie-Ange Mouret-Reynier

**Affiliations:** 1 INSERM U1240 Imagerie Moléculaire et Stratégies Théranostiques (IMoST), Université Clermont Auvergne, Clermont-Ferrand, F- 63000 France; 2 Centre d’Investigation Clinique UMR501, Clermont-Ferrand, F-63000 France; 3 Département de Recherche Clinique, Délégation Recherche Clinique et Innovation, Centre Jean PERRIN, Clermont-Ferrand, F-63000 France; 4 Service d’oncologie médicale, Centre Jean PERRIN, Clermont-Ferrand, F-63000 France; 5 Service d’oncologie médicale, Centre Eugène MARQUIS, Rennes, France; 6 Service d’oncologie médicale, Institut de Cancérologie Strasbourg Europe, Strasbourg, France; 7 Service d’oncologie médicale, Institut du Cancer de Montpellier Val d’Aurelle, Montpellier, France; 8 Service d’oncologie médicale, Institut de Cancérologie de l’Ouest, René GAUDUCHEAU, Saint Herblain, France; 9 Service d’oncologie médicale et radiothérapie, Clinique Tivoli, Bordeaux, France; 10 Service d’oncologie médicale, CHU Dupuytren, Limoges, France; 11 Service d’oncologie médicale, Centre François BACLESSE, Caen, France; 12 Service d’oncologie médicale, Institut Gustave ROUSSY, Villejuif, France; 13 Service d’oncologie médicale, Centre Hospitalier de Blois, Blois, France; 14 Service d’oncologie médicale, Institut Jean GODINOT, Reims, France; 15 Service d’oncologie médicale, Centre Hospitalier de Pau, Pau, France; 16 Service d’anatomopathologie, Centre Jean PERRIN, Clermont-Ferrand, France; 17 Unicancer, Paris, France; 18 Centre d’oncologie, Université King Saud (Medical City), Riyadh, Arabi Saoudite

**Keywords:** HER2-positive breast Cancer (HER2 + BC), TOP2A, Trastuzumab, Pertuzumab, Pathological complete response (pCR)

## Abstract

**Purpose:**

Previous studies have reported the benefit of dual HER2-targeting combined to neoadjuvant chemotherapy in HER2-amplified breast cancer (HER2 + BC). Moreover, besides the cardiac toxicity following their association to Trastuzumab, anthracyclines chemotherapy may not profit all patients. The NeoTOP study was designed to evaluate the complementary action of Trastuzumab and Pertuzumab, and the relevance of an anthracycline-based regimen according to TOP2A amplification status.

**Methods:**

Open-label, multicentre, phase II study. Eligible patients were aged ≥ 18 with untreated, operable, histologically confirmed HER2 + BC. After centralized review of TOP2A status, TOP2A-amplified (TOP2A+) patients received FEC100 for 3 cycles then 3 cycles of Trastuzumab (8 mg/kg then 6 mg/kg), Pertuzumab (840 mg/kg then 420 mg/kg), and Docetaxel (75mg/m^2^ then 100mg/m^2^). TOP2A-not amplified (TOP2A-) patients received 6 cycles of Docetaxel (75mg/m^2^) and Carboplatin (target AUC 6 mg/ml/min) plus Trastuzumab and Pertuzumab. Primary endpoint was pathological Complete Response (pCR) using Chevallier’s classification. Secondary endpoints included pCR (Sataloff), Progression-Free Survival (PFS), Overall Survival (OS), and toxicity.

**Results:**

Out of 74 patients, 41 and 33 were allocated to the TOP2A + and TOP2A- groups respectively. pCR rates (Chevallier) were 74.4% (95%CI: 58.9–85.4) vs. 71.9% (95%CI: 54.6–84.4) in the TOP2A + vs. TOP2A- groups. pCR rates (Sataloff), 5-year PFS and OS were 70.6% (95%CI: 53.8–83.2) vs. 61.5% (95%CI: 42.5–77.6), 82.4% (95%CI: 62.2–93.6) vs. 100% (95%CI: 74.1–100), and 90% (95%CI: 69.8–98.3) vs. 100% (95%CI: 74.1–100). Toxicity profile was consistent with previous reports.

**Conclusion:**

Our results showed high pCR rates with Trastuzumab and Pertuzumab associated to chemotherapy. They were similar in TOP2A + and TOP2A- groups and the current role of neoadjuvant anthracycline-based chemotherapy remains questioned.

**Trial registration number:**

NCT02339532 (registered on 14/12/14).

## Introduction

Breast Cancer (BC) is the most prevalent cancer worldwide with 2.26 million cases in 2020 according to the World Health Organization (WHO). BC is a heterogeneous disease which encompasses a large number of molecular subtypes. The survival rates among patients differ based on the molecular subtype and stage. Fifteen to 20% of BC report an overexpression or amplification of the Human Epidermal growth factor Receptor 2 (HER2/ERBB2) [1]. HER2 positive (HER2+) tumours have historically been associated with a worse prognosis than other BC due to their aggressiveness and shortened disease-free and overall survivals [1]. However, the development of Trastuzumab (Herceptin®), a humanized monoclonal antibody targeting HER2, revolutionized their treatment in the metastatic and in the adjuvant setting [2–5]. In the neoadjuvant setting, when associated with chemotherapy, Trastuzumab proved to be more effective than chemotherapy alone with a significantly higher pathologic Complete Response (pCR) rate (50% versus 32%), a longer survival (25.1 months versus 20.3 months) and time to disease progression (7.4 months versus 4.6 months), and a lower rate of death (22% versus 33%) [6–9]. Other anti HER2 agents have been developed thereafter with complementary actions by binding different sites of HER2. The benefit of Pertuzumab combined to Trastuzumab and chemotherapy was shown in the NeoSphere and TRYPHAENA trials [10, 11]. While Trastuzumab leads to HER2 internalisation, Pertuzumab prevents its dimerization [12, 13], and the addition of Pertuzumab to Trastuzumab and Docetaxel led to a significant improvement of pCR when compared to Trastuzumab and Docetaxel alone in HER2 + tumours (46% versus 29%) [10].

The topoisomerase 2 A (TOP2A) gene is located on chromosome 17q12-q21, near HER2 locus. Previous reports have indicated that TOP2A gene aberrations, amplification or deletion, are seen in connection with HER2 + BC [14, 15]. Moreover, TOP2A amplification was linked with an improved chemosensitivity to anthracycline-based regimen [16–19]. A large retrospective analysis in the metastatic setting highlighted that patients with HER2/TOP2A co-amplification and treated with anthracycline-containing regimen have a longer survival compared with HER2-positive patients lacking TOP2A amplification [17]. Conversely, preclinical studies have shown that TOP2A deletion results in reduced expression of TOP2A proteins, and hence reduced sensitivity to chemotherapy (fewer drug targets available) [20, 21]. Approximately 37% of BC display HER2+/TOP2A + coamplification [15, 17, 22]. TOP2A gene encodes the Deoxyribonucleic Acid (DNA) Topoisomerase II alpha, a gyrase involved in DNA replication, transcription and disentanglement. As a direct target of anthracyclines, TOP2A is a potential predictive factor to anthracycline-based therapy response [23, 24]. This could allow to reserve this potentially cardiotoxic chemotherapy to the patients who can get additional benefit from it, cardiotoxicity being even more important in HER2 tumors due to the concomitant potential cardiotoxicity of anti HER2 agents.

The NeoTOP study was designed to evaluate the neoadjuvant strategy for HER2 + tumors, in terms of pCR, based on the results of the TOP2A analysis, the chemotherapy backbone of the treatment being adapted according to the status of TOP2A in these tumors. We report here for the first time the outcome and the toxicity results of both strategies.

## Patients and methods

### Study design and participants

NeoTOP was a prospective, multicenter, open-label, non-randomized phase II trial conducted in 13 centres in France (NCT02339532). Eligible patients were 18 years or older women with untreated, operable, HER2 + stage I and IIa, clinically > 1 cm tumor, any lymph node status, non-metastatic (M0) BC. Hormone Receptor had to be negative (HR-) for T1c tumor, otherwise any HR was accepted WHO performance status had to be 0 or 1. Prior to inclusion, a mandatory centralized review of HER2 and TOP2A status was performed. According to the TOP2A status, patients were allocated to either TOP2A amplified (TOP2A+) or not amplified (TOP2A-) groups.

### Treatment procedure

**Fig. 1:**
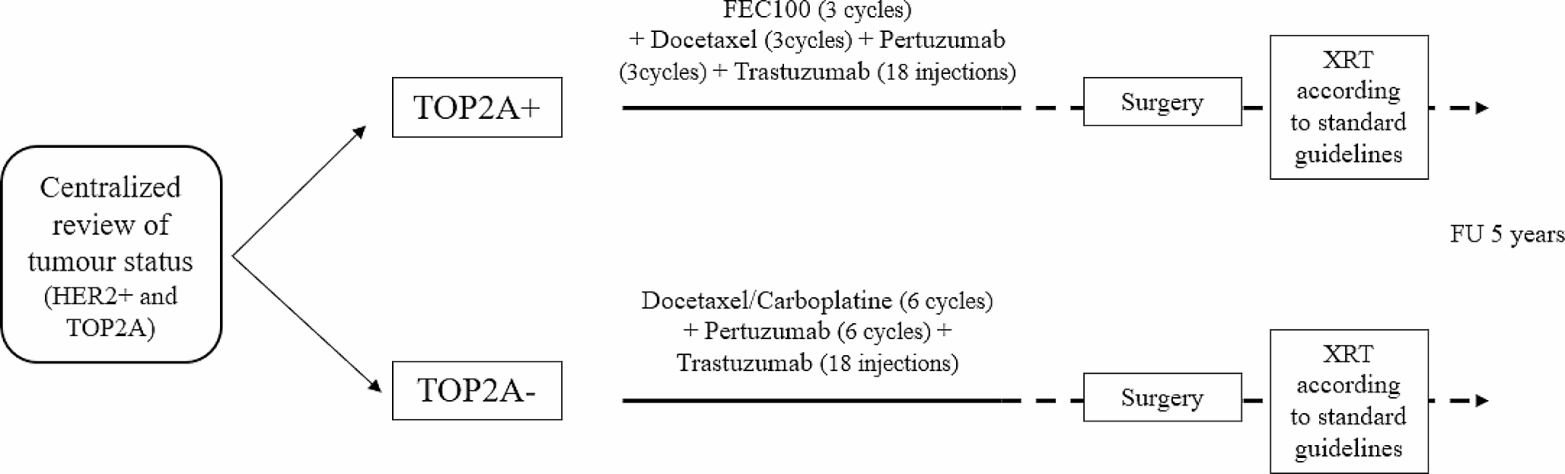
Study design

The mutational status of TOP2A was assessed at the Jean Perrin Center, Clermont-Ferrand, France, using fluorescence in situ hybridization (FISH) (ratio > 2,2). Patients were scheduled to receive 6 cycles of neoadjuvant chemotherapy. In the TOP2A + group, the anthracycline-based chemotherapy consisted of FEC 100 (5-Fluorouracil 500mg/m^2^, Epirubicin 100mg/m^2^, Cyclophosphamide 500mg/m^2^) for 3 cycles followed by 3 cycles of Trastuzumab (8 mg/kg at cycle 4 then 6 mg/kg at cycles 5 and 6; IV), Pertuzumab (840 mg/kg at cycle 4 then 420 mg/kg at cycles 5 and 6; IV), and Docetaxel (75mg/m^2^ at cycle 4 then 100mg/m^2^ at cycles 5 and 6; IV). In the TOP2A- group, chemotherapy consisted of 6 cycles of Docetaxel (75mg/m^2^; IV) and Carboplatin (target AUC 6 mg/ml/min; IV) associated with Trastuzumab (loading dose of 8 mg/kg then 6 mg/kg; IV) and Pertuzumab (loading dose of 840 mg/kg then 420 mg/kg; IV). In both groups, Trastuzumab was continued as adjuvant treatment and patients received a total of 18 infusions (Fig. 1).

### Assessments

The primary endpoint was the pCR rates evaluated in the TOP2A + and TOP2A- groups using the Chevallier’s classification [25]. A pCR was defined by the absence of invasive cancer cells in both breast tissues and lymph nodes (grades 1 and 2 of the Chevallier’s classification). The pCR was centrally reviewed.

The secondary endpoints were (1) pCR, measured using the Sataloff’s classification, pCR was then defined by total or near total therapeutic effect (tumour response T-A) or evidence of therapeutic effect and no metastatic disease (lymph node response N-A), or no therapeutic effect but no metastasis (lymph node response N-B) (Sataloff et al., 1995). (2) Progression-Free Survival (PFS) defined as the time from the first administration of treatment to progression or death from any cause (if progression had not been documented). (3) Overall Survival (OS), defined as the time from the first administration of treatment to death from any cause. (4) the rate of breast conserving surgery. (5) the radiological response assessed on mammography and breast echography before, during (after two cycles of treatment), and after the end of treatment (before surgery). For the latter, bidimensional measurements were used to estimate tumour size between cycle 3 of treatment and surgery according to the WHO criteria. Responses were classified as follows: complete response (CR; disappearance of all target lesions), partial response (PR; >50% reduction in cross product), stable disease (SD; < 50% reduction or < 25% increase in cross product) and progressive disease (PD; > 25% increase in the cross product or any new lesion). The overall safety profile of the treatment was determined by the occurrence of Adverse Events (AEs). Toxicities were assessed every week from first study drugs intake up to 30 days after the last dose was taken. Severity was graded according to the National Cancer Institute Common Terminology Criteria for Adverse Events version 4.0 (NCI CTCAE v4.0). Paraclinical examination were done for the assessment of cardiac function according to recommendations: left ventricular ejection fraction (LVEF) at D15-21 of cycle 3 and every three months during adjuvant trastuzumab treatment, then every 6 months from the last dose of trastuzumab to 24 months and every year during 5 years after the last dose of trastuzumab or more if a sustained decrease is observed and electrocardiograms were performed at cycle 4 and at post-treatment visit.

### Statistical analysis

The study used a three-stage Fleming design, allowing early termination either for futility or efficacy, separately in each group (TOP2A+, and TOP2A-), and sample size was chosen following Fleming’s initial procedure [26]. Based on previous studies such as NEOSPHERE [10], TRYPHAENA [11], and BCIRG-006 trials [5], in the TOP2A amplification group, the minimal rate of efficacity for the Fleming design was set at 60%, and the maximal rate of inefficacy at 40%. It was determined that for a one-stage procedure, a sample size of 37 would be necessary to achieve 80% power with 5% one-sided type I error rate. Following Fleming’s guidelines, it was chosen to use equal sample sizes for each stage, set at 15, for a total of 45 subjects, in order to account for possible attrition. In the group without TOP2A amplification, the thresholds for acceptance and rejection were set, respectively, at 55% and 35%, which yielded the same sample sizes of 15 patients per stage. Accrual was suspended for each interim analysis when both groups reached its required sample size. The study was stopped for efficacy at the second stage. Since TOP2A status, and thus group assignation, was determined after enrollment, the total number of patients included in the study at the end of the second and final stage was 86.

Statistical analysis was conducted using R software (version 4.1.0; R Core Team, 2021). The study being non-comparative, analysis was performed in each group separately. Patients, disease characteristics and received treatment were presented by absolute and relative frequencies for categorical variables, and by the mean and standard deviation (SD) or median and interquartile interval (IQI) for continuous variables. Point estimations were reported with 95% Confidence Intervals (95% CI). AEs were summarized using the max-grade method. Kaplan-Meier method was used for survival analysis, and CI for survival rates were computed using the beta product confidence procedure.

## Results

### Patients’ characteristics

**Fig. 2:**
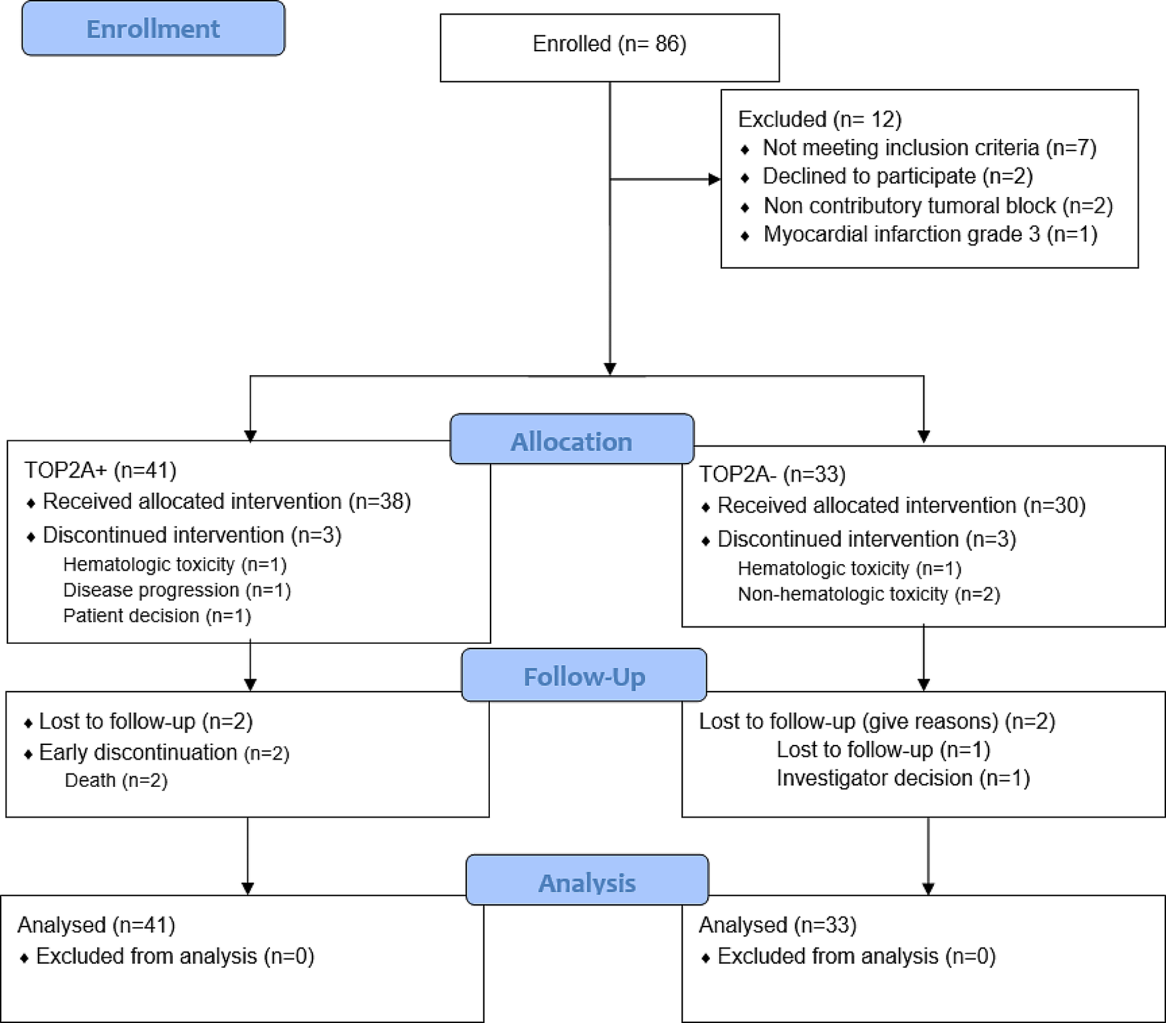
Consort diagram

Between January 2015 and December 2018, 86 patients were enrolled. The median follow-up duration was 40 months. Out of *N* = 86 patients included in the NeoTOP study, *N* = 12 patients were excluded from the analysis because they did not receive any cycle of neoadjuvant chemotherapy. TOP2A was classified as amplified in *N* = 41 patients and not amplified in *N* = 33 patients (Fig. 2).

A majority (*N* = 68; 91.9%) of patients were still alive at the end of the protocol, and *N* = 6 (8.1%) had stopped the study following death due to disease progression (*N* = 2; 2.7%), lost of follow-up (*N* = 3; 4.1%), and physician’s decision (*N* = 1; 1.4%). Baseline main characteristics of the 74 analyzed patients are detailed in Table 1. Median age was 50 years (IQI: 43–57), and WHO performance status was 0 for *N* = 70 patients (95.9%) and 1 for *N* = 3 patients (4.1%). The most common histological type was ductal (*N* = 70 patients; 94.6%). They was a higher proportion of menopausal women in the TOP2A + group (53.7%) when compared to the TOP2A- group (30.3%). The mean largest diameter of the primary tumour was 33 mm for the entire population and there were *N* = 14 (18.9%) patients with T1_C_ tumours (*N* = 5 TOP2A + and *N* = 9 TOP2A-), and *N* = 57 (77.0%) patients with T2-3 tumours (*N* = 34 TOP2A + and *N* = 23 TOP2A-). The majority of patients (*N* = 47; 63.5%) did not have clinically involved adenopathy (N0) at inclusion (*N* = 23 TOP2A + and *N* = 24 TOP2A-). Positive oestrogen and progesterone receptor status were respectively reported in 38 (51.4%) and 29 (39.2%) patients. Overall, TOP2A + and TOP2A- groups were well balanced.

**Table 1:** Patients’ baseline characteristics

Characteristics	Study population *N* = 74 (%)	TOP2A + group *N* = 41 (%)	TOP2A- group *N* = 33 (%)
**Age (years) (median, IQI)**	50 (43–57)	53 (46–59)	46 (41–52)
**WHO performance status**			
0	70 (95.9)	37 (92.5)	33 (100)
1	3 (4.1)	3 (7.5)	0 (0)
**Menopausal status (%)**			
Non-menopausal	41 (55.4)	19 (46.3)	22 (66.7)
Menopausal	32 (43.2)	22 (53.7)	10 (30.3)
Unknown	1 (1.4)	0 (0.0)	1 (3.0)
**cT (%)**			
0–1	2 (2.7)	2 (4.9)	0 (0.0)
1c	14 (18.9)	5 (12.2)	9 (27.3)
2–3	57 (77.0)	34 (82.9)	23 (69.7)
X	1 (1.4)	0 (0.0)	1 (3.0)
**cN (%)**			
0	47 (63.5)	23 (56.1)	24 (27.7)
1–3	25 (33.8)	16 (39.0)	9 (27.3)
X	2 (2.7)	2 (4.9)	0 (0)
**Histological type (%)**			
Ductal	70 (94.6)	40 (97.6)	30 (90.9)
Other	4 (5.4)	1 (2.4)	3 (9.1)
**Eostrogen Receptor status (%)**			
Negative	36 (48.6)	20 (48.8)	16 (48.5)
Positive	38 (51.4)	21 (51.2)	17 (51.5)
**Progesteron Receptor status (%)**			
Positive	45 (60.8)	24 (58.5)	21 (63.6)
Negative	29 (39.2)	17 (41.5)	12 (36.4)
Clinical size (SD*)			
Mean (mm)	33 (14.1)	35 (15.3)	32 (12.3)

*SD = Standard Deviation

### Treatments compliance

Description of the treatments received by the NeoTOP patients is detailed in Table 2. During the neoadjuvant therapy, in the TOP2A + group, 2.4% (*N* = 1) patients did not receive 5-Fluorouracil, and 7.2% (*N* = 3) had an interruption of treatment (*N* = 1 for progressive disease, *N* = 1 withdrawal by subject, *N* = 1 for AE), among which 4.9% (*N* = 2) did not receive HER2-targeted therapy. Two (4.9%) patients received one supplementary cycle of treatment (either 1 cycle of targeted-HER2 therapy combined with Docetaxel or 1 cycle of targeted-HER2 therapy alone). In the TOP2A- group, *N* = 2 (9.1%) patients did not receive Carboplatin for 1 cycle, *N* = 1 (3.0%) did not receive Carboplatin and Docetaxel for 1 cycle (for extravasation, allergy, and non-hematologic toxicity, respectively), and *N* = 3 (9.1%) patients had an interruption of treatment (*N* = 1 after 2 cycles and *N* = 2 after 5 cycles for AE). *N* = 1 (4.9%) patient received 1 supplementary cycle of targeted-HER2 therapy alone. The number of breast-conserving surgery was high in both groups but the patients allocated in the TOP2A + group were more likely to have less tissue removed during surgery with 71.8% in TOPA2 + and 53.1% in TOPA2- who had tumorectomy compared to 15.4% in TOPA2 + and 31.2% in TOPA2- who had quadrantectomy even though the difference was not significant. *N* = 1 (3.1%) patient in the TOP2A- group had second surgery. In the adjuvant setting, around half of the patients (*N* = 35; 48.6%) received a combination of HER2 + targeted-therapy and radiotherapy, while the other half (*N* = 36; 50.0%) received the same combination associated to hormonotherapy. In the TOP2A-group, *N* = 1 (3.0%) patient did not receive adjuvant Trastuzumab but chemotherapy.

**Table 2:** Description of the NeoTOP study treatments

		ALL	TOP2A+	TOP2A-
**Neoadjuvant**	**nb of cycles**	***N*** = 74	***N*** = 41	***N*** = 33
**Chemotherapy** **+ HER2+-targeted**	2	2 (2.7%)	1 (2.4%)	1 (3.0%)
	3	1 (1.4%)	1 (2.4%)	0 (0.0%)
	5	3 (4.1%)	1 (2.4%)	2 (6.1%)
	6	65 (87.8%)	36 (87.8%)	29 (87.9%)
	7	3 (4.1%)	2 (4.9%)	1 (3.0%)
**Reason for treatment interruption**		*N* = 6	*N* = 3	*N* = 3
**Toxicity**				
**Hematologic toxicity**		2 (2.7%)	1 (33.3%)	1 (33.3%)
**Non-hematologic toxicity**		2 (2.7%)	0 (0.0%)	2 (66.7%)
**Progressive disease**		1 (1.4%)	1 (33.3%)	0 (0.0%)
**Patient decision**		1 (1.4%)	1 (33.3%)	0 (0.0%)
**Surgery type**		**N = 71**	**N = 39**	**N = 32**
**Masectomy**		10 (14.1%)	5 (12.8%)	5 (15.6%)
**Quadrantectomy**		16 (22.5%)	6 (15.4%)	10 (31.2%)
**Tumorectomy**		45 (63.4%)	28 (71.8%)	17 (53.1%)
**Sentinel node**		**N = 70**	**N = 39**	**N = 31**
**No**		36 (51.4%)	18 (46.2%)	18 (58.1%)
**Yes**		34 (48.6%)	21 (53.8%)	13 (41.9%)
**Axillary clearance**		**N = 71**	**N = 39**	**N = 32**
**No**		35 (49.3%)	20 (51.3%)	15 (46.9%)
**Yes**		36 (50.7%)	19 (48.7%)	17 (53.1%)
**Second surgery**		**N = 71**	**N = 39**	**N = 32**
**No**		70 (98.6%)	39 (100.0%)	31 (96.9%)
**Yes**		1 (1.4%)	0 (0.0%)	1 (3.1%)
**Adjuvant**		*N* = 72	*N* = 39	*N* = 33
HER2+-targeted therapy + Radiotherapy		35 (48.6%)	20 (51.3%)	15 (45.5%)
HER2+-targeted therapy + Hormonotherapy + Radiotherapy		37 (51.4%)	19 (48.7%)	18 (54.5%)

### Efficacy results

**Fig. 3:**
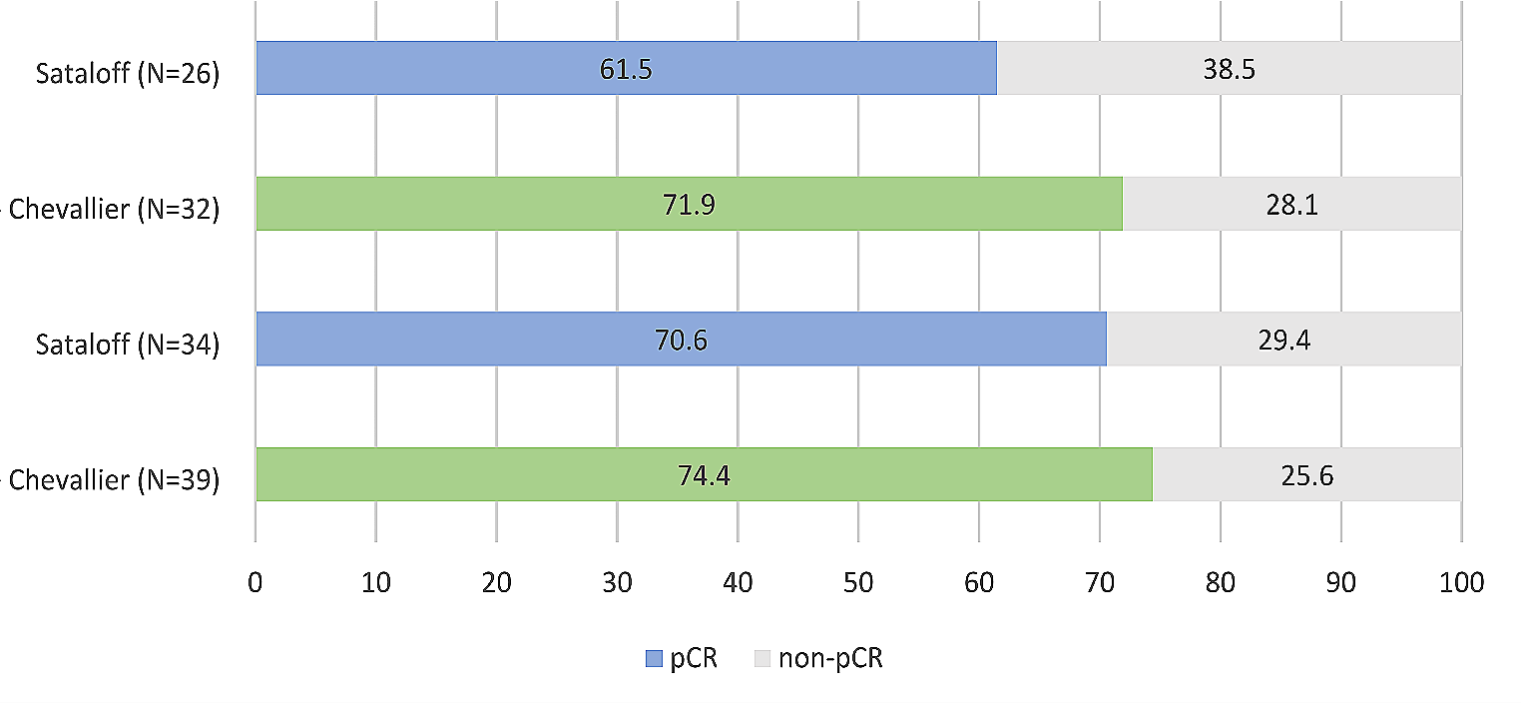
Assessment of pCR in TOP2A + and TOP2A- groups

pCR rates were 74.4% (95%CI: 58.9–85.4) vs. 71.9% (95%CI: 54.6–84.4) with 29 vs. 23 complete responses, 10 vs. 9 non-pCR, and 2 vs. 1 missing data in TOP2A + vs. TOP2A- groups (Fig. 3). Secondary endpoint included pCR evaluated with the Sataloff method, OS, and radiological response measured before, during (at C3) and after treatment. 14 patients were not evaluable for response using the Sataloff classification. Response was consequently determined in 60 patients (Fig. 3).

**Fig. 4:**
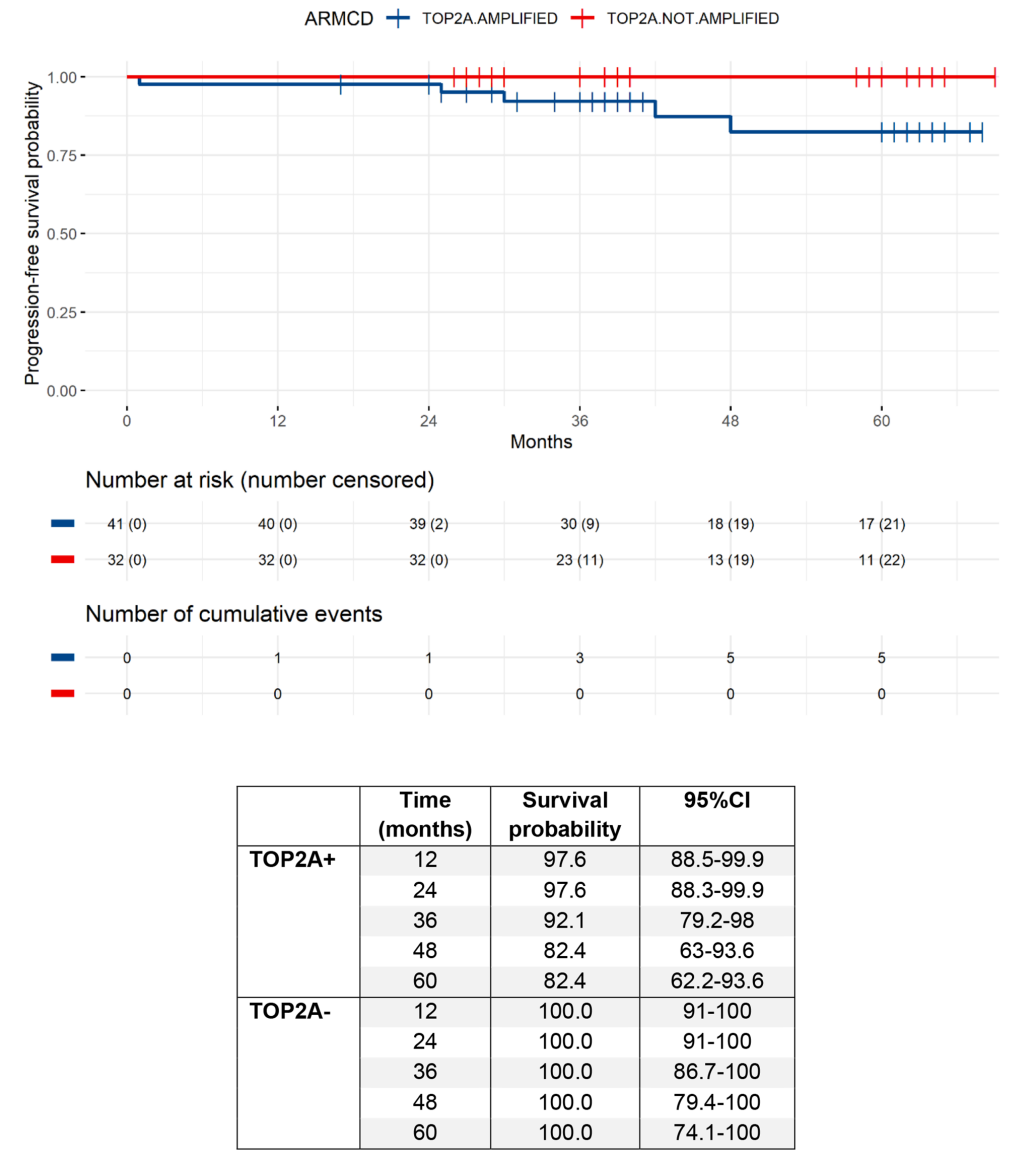
PFS in the TOP2A + and TOP2A- groups

Twenty-four vs. 16 had a complete response (pCR if grade TA-NA or TA-NB), allowing pCR rates of 70.6% (95%CI: 53.8–83.2) vs. 61.5% (95%CI: 42.5–77.6) in TOP2A + vs. TOP2A- groups. By the time of data cut-off, median follow-up was 40 months (min-max: 17–69). Five (6.8%) PFS events were reported in the study population. Five-year PFS rate was 82.4% (95% CI: 62.2–93.6) for the TOP2A + group and 100% (95% CI: 74.1–100) for the TOP2A- group (Fig. 4).

**Fig. 5:**
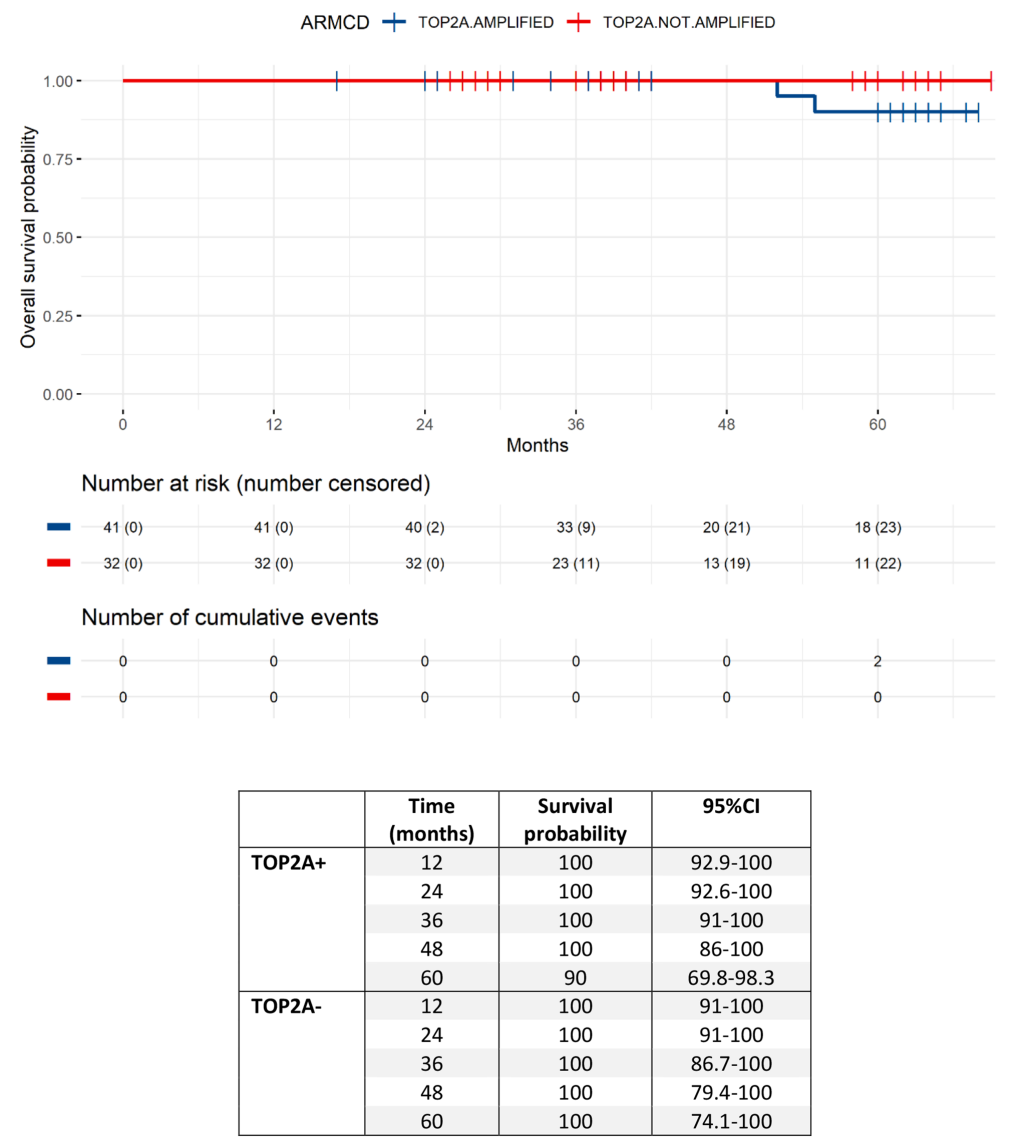
OS in the TOP2A + and TOP2A- groups

In total, OS events had been observed in 2 patients (2.7%), both patients died from disease progression. Five-year OS rate was 90% (95% CI = 69.8–98.3) for the TOP2A + group and 100% (95% CI: 74.1–100) for the TOP2A- group (Fig. 5).

Finally, physical examination measurements determined 13 (21.3%) and 39 (67.2%) CR at C3 and surgery, respectively, 6 (9.8%) and 1 (1.7%) PR, 6 (9.8%) and 1 (1.7%) SD, and 36 (59%) and 17 (29.3%) were not evaluable (comparison with all previous lesions not feasible) but did not progress. Using imaging modalities, early CR were observed in 5 patients (7.9%) evaluated by echography and in 9 (15.3%) evaluated by mammography, whereas late CR were observed in 19 (31.7%) women evaluated by echography and 20 (33.3%) patients evaluated by mammography. No signs of new lesions were detected either by physical examination or by imaging evaluation at C3 or post-treatment (data not shown). Physical and radiologic responses data using imaging modalities and according to TOP2A amplification are summarized in Table 3.

**Table 3:** Physical and imaging response assessments during (at C3) and post-neoadjuvant treatment

	level	ALL		TOP2A+		TOP2A-	
		C3*	Post-TT*	C3*	Post-TT*	C3*	Post-TT*
**CLINICAL RESPONSE**		***N*** = 61	***N*** = 58	***N*** = 36	***N*** = 34	***N*** = 25	***N*** = 24
	CR**	13 (21.3)	39 (67.2)	5 (13.9)	22 (64.7)	8 (32.0)	17 (70.8)
	PR***	6 (9.8)	1 (1.7)	5 (13.9)	0 (0.0)	1 (4.0)	1 (4.2)
	**ST** ^**#**^	6 (9.8)	1 (1.7)	2 (5.6)	1 (2.9)	4 (16.0)	0 (0.0)
	**NE** ^**##**^	36 (59.0)	17 (29.3)	24 (66.7)	11 (32.4)	12 (48.0)	6 (25.0)
**ECHOGRAPHY RESPONSE**		***N*** = 63	***N*** = 60	***N*** = 36	***N*** = 34	*N* = 27	***N*** = 26
	CR**	5 (7.9)	19 (31.7)	1 (2.8)	9 (26.5)	4 (14.8)	10 (38.5)
	PR***	20 (31.7)	13 (21.7)	11 (30.6)	8 (23.5)	9 (33.3)	5 (19.2)
	**PD** ^**###**^	1 (1.6)	1 (1.7)	1 (2.8)	1 (2.9)	0 (0.0)	0 (0.0)
	**ST** ^**#**^	18 (28.6)	5 (8.3)	13 (36.1)	3 (8.8)	5 (18.5)	2 (7.7)
	**NE** ^**##**^	19 (30.2)	22 (36.7)	10 (27.8)	13 (38.2)	9 (33.3)	9 (34.6)
**MAMMOGRAPHY RESPONSE**		***N*** = 59	***N*** = 60	***N*** = 33	***N*** = 34	***N*** = 26	***N*** = 26
	CR**	9 (15.3)	20 (33.3)	4 (12.1)	10 (29.4)	5 (19.2)	10 (38.5)
	PR***	5 (8.5)	6 (10)	3 (9.1)	3 (8.8)	2 (7.7)	3 (11.5)
	**ST** ^**#**^	13 (22.0)	4 (6.7)	10 (30.3)	2 (5.9)	3 (11.5)	2 (7.7)
	**NE** ^**##**^	32 (54.2)	30 (50)	16 (48.5)	19 (55.9)	16 (61.5)	11 (42.3)

*Post-TT: Post-neoadjuvant treatment; **CR: Complete Response; ***PR: Partial Response; ^#^ST: Stable disease; ^##^NE: not evaluable; ^###^PD: Progressive Disease

### Toxicity assessment

All patients were assessed for toxicities during the treatment and up to 30 days after the last dose intake (Table 4). The most frequently reported grade (G) ≥ 3 treatment-related adverse events (TRAEs) were hematological: 16 (39.0%) vs. 6 (18.2%) neutropenia, 5 (12.2%) vs. 0 (0.0%) leukopenia, 1 (2.4%) vs. 4 (12.1%) thrombocytopenia, and 2 (4.9%) vs. 2 (6.1%) lymphopenia in groups TOP2A + and TOP2A-, respectively. Non-hematological G ≥ 3 TRAEs mainly included 3 (7.3%) and 4 (12.1%) diarrhoea in groups TOP2A + and TOP2A-, respectively (Table 3). In total, 12 (16.2%) treatment-related serious AEs (SAEs) were reported, predominantly blood and lymphatic system disorders (5.4% neutropenia and 5.4% febrile neutropenia) (Table 5). There was no treatment-related mortality and, no increase of cardiotoxicity was observed compared to previous reports. Concerning cardiac function assessment, a decrease of LVEF grade 2 was observed.

**Table 4:** Grade ≥ 3 treatment-related adverse events (TRAEs)

Name	Grade max.	ALL n (%)	TOP2A+ n (%)	TOP2A-n (%)
NAUSEA	3	2 (2.7)	2 (4.9)	0 (0.0)
ANEMIA	3	3 (4.1)	0 (0.0)	3 (9.1)
DIARRHOEA	3	7 (9.5)	3 (7.3)	4 (12.1)
NEUTROPENIA	3	14 (18.9)	10 (24.4)	4 (12.1)
	4	8 (10.8)	6 (14.6)	2 (6.1)
ASTHENIA	3	3 (4.1)	2 (4.9)	1 (3.0)
LEUKOPENIA	3	5 (6.8)	5 (12.2)	0 (0.0)
FATIGUE	3	3 (4.1)	2 (4.9)	1 (3.0)
MUCOSAL INFLAMMATION	3	3 (4.1)	3 (7.3)	0 (0.0)
THROMBOCYTOPENIA	3	4 (5.4)	1 (2.4)	3 (9.1)
	4	1 (1.4)	0 (0.0)	1 (3.0)
GAMMA GLUTAMYLTRANSFERASE INCREASED	3	1 (1.4)	1 (2.4)	0 (0.0)
LYMPHOPENIA	3	4 (5.4)	2 (4.9)	2 (6.1)
VOMITING	3	1 (1.4)	1 (2.4)	0 (0.0)
STOMATITIS	3	2 (2.7)	2 (4.9)	0 (0.0)
LYMPHOPENIA	3	1 (1.4)	1 (2.4)	0 (0.0)

**Table 5:** Grade ≥ 3 treatment-related serious adverse events (SAEs)

Name	Grade max.	ALL n (%)	TOP2A+ n (%)	TOP2A-n (%)
NEUTROPENIA	4	4 (5.4)	3 (42.9)	1 (8.3)
FEBRILE NEUTROPENIA	3	2 (2.7)	0 (0)	2 (16.7)
	4	2 (2.7)	0 (0)	2 (16.7)
FEBRILE BONE MARROW APLASIA	3	1 (1.4)	1 (14.3)	0 (0)
	4	1 (1.4)	0 (0)	1 (8.3)
DIARRHOEA	3	1 (1.4)	0 (0)	1 (8.3)
EXTRAVASATION	3	1 (1.4)	0 (0)	1 (8.3)

## Discussion

The phase 2 NeoTOP study was designed to assess the interest of dual HER2 targeting in the neoadjuvant setting combined with anthracycline-based or anthracycline-free chemotherapy according to TOP2A status in women with operable HER2 + BC. The results presented herein were consistent with previous findings demonstrating a high pCR rate associated with the use of Trastuzumab plus Pertuzumab in combination with neoadjuvant chemotherapy [10]. In the whole population, a total of *N* = 52 patients achieved pCR (73.2% [95%CI: 61.9–82.2]). Interestingly, only *N* = 1 patient was classified as a grade 4 using the Chevallier’s classification, indicating a high chemosensitivity, even non-pCR. The complementary dual-blockade of the HER2 receptor may have contributed to obtain such a high rate, which was linked to more breast conserving surgery (85.9%; *N* = 61 patients) than mastectomy (14.1%; *N* = 10 patients). As demonstrated by the FDA-meta-analysis or the NeoALTTO trial [27, 28], pCR is correlated with favourable patient outcome, particularly in hormone-negative HER2 + early BC, and accordingly, with only *N* = 5 (6.8%) patients who experienced a disease recurrence and *N* = 2 (2.7%) who died of disease progression, 5-year PFS and OS rates were respectively 89.5% [95%CI: 75.7–96.4] and 93.9% [95%CI: 80.4–99] in the NeoTOP population, with a median follow-up of 40 months (min-max: 17–69).

Since 2017, the St Gallen consensus meeting have clearly highlighted the neoadjuvant approach as the preferred treatment option in HER2 + BC equal or larger than 2 cm or with axillary lymph node involvement [29]. However, if neoadjuvant chemotherapy is appropriate for many women with BC, treatment with Trastuzumab has been associated with cardiac dysfunction, especially when combined with anthracyclines [5, 30]. Moreover, a response allowing both less extensive surgery and improved surgical outcomes may be possible in an important subset of patients with reduced doses of chemotherapy [31]. In the NeoSphere trial, 17% of the patients achieved pCR after they were given Trastuzumab plus Pertuzumab only, suggesting that not all patients require chemotherapy [10]. In this context, the NeoTOP study was designed to investigate the relevance of anthracycline-containing chemotherapy based on the amplification status of the TOP2A gene. TOP2A is a direct target and as such, a potential marker to predict response to anthracycline-based therapy. Our analyses did not provide any additional information supporting TOP2A predictive value in HER2 + BC. In fact, in the NeoTOP population, the benefit of Trastuzumab plus Pertuzumab combined with chemotherapy was maintained regardless of TOP2A status with similar pCR rates in TOP2A + and TOP2A- groups. There was also no significant difference between the two groups in terms of 5-year PFS and 5-year OS. This absence of impact may be explained by the size of TOP2A amplification which may vary from one individual to another however biomarkers analysis conducted in TRYPHAENA study did not show any association between TOP2A expression levels and pCR rate according to anthracycline-containing or anthracycline-free regimen [11]. Another hypothesis may be that the target of anthracyclines is the protein, not the gene, and gene amplification does not necessarily translate into mRNA and protein expression. In that regard, regulatory mechanisms playing roles in regulating mRNA and protein abundance, such as transcription factors, miRNA, DNA methylation, and other translational and post-translational modifications as well as protein stability, should be taken into consideration in future studies aiming to ascertain TOP2A prognostic significance in HER2 + BC [32–35]. In the NeoTOP trial, the correlation between TOP2A gene amplification and protein level of expression will be further explored by immunohistochemistry using the Formalin-Fixed Paraffin-Embedded (FFPE) tumour tissue samples collected at different time points during the trial. Additionally, the standard tools and cut-off values for estimating TOP2A status remains to be standardized, especially as TOP2A amplification may be associated with tumour size and stage [36].

Regarding safety, our data are also in agreement with previous reports [5, 30]. In the NeoTOP trial, the association of Trastuzumab plus Pertuzumab in combination with chemotherapy was well tolerated. with similar hematotoxicity as previously reported. No new safety events were reported. The two deaths were due to the progression of the disease and, more importantly, NeoTOP patients did not experience cardiac toxicity. The CLEOPATRA study previously demonstrated that the addition of Pertuzumab to Trastuzumab does not significantly increase cardiac toxicity, and the long observation period allowed to exclude any further risk from this addition [37]. A large recent meta-analysis showed that there are no difference in efficacy between patients treated with anti-HER2-targeted neoadjuvant therapy combined with or without anthracyclines but LVEF decrease is significantly lower in patients treated with anti-HER2-targeted therapy and anthracycline-free regimen [38]. It’s important to keep in mind that the use of anthracycline for HER2-positive BC varies by country. The National Comprehensive Cancer Network guideline (v4.2021) and the Chinese Society of Clinical Oncology guideline (2020 version) favor an anthracycline-free regimen [39, 40].

Some important limitations of the NeoTOP study have to be underlined. The main limitation being the low number of assessable patients. NeoTOP underlines the limitations of clinical examination to objectively and precisely evaluate response. Indeed, based on clinical assessment at surgery, 39 patients (67.2%) were considered as complete clinical responder, while 52 (73.2%) had pCR. Using imaging modalities, 19 (31.7%) and 20 (33.3%) women were considered CR by echography and mammography. However, a limited number of patients had evaluable data since 17, 22, and 30 patients out of 74 were not evaluable for clinical examination, echography and mammography, respectively. Thus, the analyses focus on relatively small subgroups of patients, and this may have hampered the possibility to obtain significant conclusions. The study focused on lower risk patient (stage I and IIa tumors), that can be seen as a limitation.

To summarize, the NeoTOP study added to the large body of evidence proving the utility of combining different neoadjuvant treatment modalities to develop the best treatment options for patients with HER2 + BC [41, 42]. Additional studies will be required to fully investigate the predictive role of TOP2A in response to anthracyclines-based regimen as it still remains unclear whether TOP2A overexpression arise from gene amplification. Furthermore, in view of the similar pCR rates recorded in both groups and the frequency of the haematological toxicities observed in both groups, the omission of anthracyclines from neoadjuvant treatment might be beneficial for some patients. The identification of predictive factors is a key requirement to select successful treatments and refine patient selection, new markers will be investigated using the NeoTOP data and samples to characterize response to anthracycline-based or anthracycline-free treatments.

## Data Availability

The datasets generated during and/or analyzed during the current study are not publicly available due to information that could compromise research participant consent. According to French and European regulations, any re-use of the data must be approved by the ethical committee CPP Sud-Est VI. Each request for access to the dataset (including the images) will be granted upon reasonable request sent to the corresponding author.

## Abbreviations

AE: Adverse event
AUC: Area under curve
BC: Breast cancer
CI: Confidence intervals
CR: Complete response
FEC: 5-Fluorouracil, Epirubicin, Cyclophosphamide
FISH: Fluorescence in situ hybridization
HER2+: HER2-amplified
IQI: Interquartile interval
NCI CTCAE: National Cancer Institute Common Terminology Criteria for Adverse Events
OS: Overall survival
pCR: Pathological Complete Response
PD: Progressive disease
PFS: Progression free survival
PR: Partial response
SD: Stable disease
SD: Standard deviation
TOP2A: Topoisomerase 2 A
TOP2A+: TOP2A amplified
TOP2A-: TOP2A not amplified
WHO: World Health Organization
